# Non-Invasive Assessment of Congestion by Cardiovascular and Pulmonary Ultrasound and Biomarkers in Heart Failure

**DOI:** 10.3390/diagnostics12040962

**Published:** 2022-04-12

**Authors:** Adriana Mihaela Ilieșiu, Andreea Simona Hodorogea, Ana-Maria Balahura, Elisabeta Bădilă

**Affiliations:** 1 Cardiology and Internal Medicine Department, Theodor Burghele Clinical Hospital, Carol Davila University of Medicine and Pharmacy, 020021 Bucharest, Romania; adriana.iliesiu@umfcd.ro; 2 Internal Medicine Department, Bucharest Clinical Emergency Hospital, Carol Davila University of Medicine and Pharmacy, 020021 Bucharest, Romania; ana-maria.balahura@umfcd.ro (A.-M.B.); elisabeta.badila@umfcd.ro (E.B.)

**Keywords:** heart failure, congestion, echocardiography, vascular ultrasound, pulmonary ultrasound, biomarkers

## Abstract

Worsening chronic heart failure (HF) is responsible for recurrent hospitalization and increased mortality risk after discharge, irrespective to the ejection fraction. Symptoms and signs of pulmonary and systemic congestion are the most common cause for hospitalization of acute decompensated HF, as a consequence of increased cardiac filling pressures. The elevated cardiac filling pressures, also called hemodynamic congestion, may precede the occurrence of clinical congestion by days or weeks. Since HF patients often have comorbidities, dyspnoea, the main symptom of HF, may be also caused by respiratory or other illnesses. Recent studies underline the importance of the diagnosis and treatment of hemodynamic congestion before HF symptoms worsen, reducing hospitalization and improving prognosis. In this paper we review the role of integrated evaluation of biomarkers and imaging technics, i.e., echocardiography and pulmonary ultrasound, for the diagnosis, prognosis and treatment of congestion in HF patients.

## 1. Background

Most of the patients hospitalized for worsening heart failure (HF), 60% in the EURObservational Research Programme of the European Society of Cardiology, have dyspnoea and signs of congestion [1]. The index-hospitalization for HF is associated with recurrent hospitalizations, 24% at 30 days and over 50% after 6 months, and one-year mortality roughly 27% [2,3]. The myocardial dysfunction worsens with each HF decompensation, leading to HF progression [1,4]. Congestion is a major cause of HF worsening regardless of the left ventricular ejection fraction (EF) [5]. Hemodynamic congestion, defined by volume overload and increased filling pressures, may precede the onset of clinical congestion by days or weeks. The risk of re-hospitalization is progressively increased when invasively measured pulmonary diastolic pressure is above 18 mm Hg [6]. Clinical congestion is characterized by progressive dyspnoea, weight gain, and signs of systemic venous congestion. 

In HF registries one in five patients hospitalized have residual clinical or hemodynamic congestion at discharge [7]. The persistence of clinical congestion is linked to unfavourable prognosis, especially in the setting of kidney dysfunction [8,9,10]. Despite improvement in clinical congestion, the higher level of natriuretic peptides (NPs) during hospitalization, as biomarkers of hemodynamic congestion, are predictors of increased morbi-mortality both at discharge, and one week post-discharge [11,12].

The clinical evaluation of congestion has many limitations. Physical examination has a low sensitivity and specificity to identify pulmonary congestion, and auscultation of the lung poorly correlates with left ventricular (LV) filling pressures [13,14,15]. Although dyspnoea improvement occurs during the first two days of treatment, it neither accurately reflects de-congestion, nor is it a predictable prognostic factor. Moreover, clinical assessment of the changes in systemic congestion has significant intra- and inter-observer variability, notable in obese patients, and a mild degree of congestion is difficult to diagnose [16,17]

All these data underline the importance of non-invasive detection of congestion in patients with HF, in order to reduce re-hospitalizations, to improve the prognosis, and to decrease mortality by optimizing HF treatment. 

Several imaging and biochemical markers have been proposed for the assessment of congestion. The ultrasonographic evaluation of the heart, veins and lung provides useful information about congestion and has been validated as prognostic markers, both in acute and chronic settings [18]. In the recent years, point of care ultrasonography (POCUS) has emerged as a promising bedside diagnostic tool performed by the treating physician to evaluate volume status and to monitor decongestive therapy. POCUS is a limited, centred bedside ultrasound examination, consisting of the focused cardiac ultrasound (FoCUS), inferior vena cava assessment, as well as lung ultrasound (LUS) to detect the presence of the extravascular fluid.

## 2. Echocardiography and Vascular Ultrasound

Echocardiography has a crucial role in the diagnosis of HF, providing structural and functional information and becoming an integral part of the clinical examination nowadays [19].

### 2.1. Left and Right Ventricular Filling Pressures

#### 2.1.1. Left Ventricular Filling Pressures and Pulmonary Congestion

**Figure 1:**
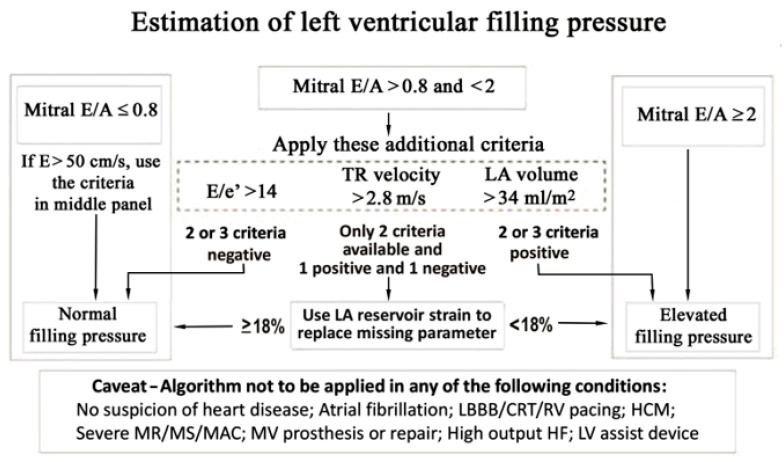
Algorithm for estimation of LV filling pressure (modified from Smiseth OA, Morris DA, Cardim N, et al. Multimodality imaging in patients with heart failure and preserved ejection fraction: an expert consensus document of the European Association of Cardiovascular Imaging. Eur. Heart J. Cardiovasc. Imaging. 2022, 23(2), e34–e61. doi:10.1093/ehjci/jeab154. PMID: 34729586. [20]). A = the late filling wave due to atrial contraction, E = the early rapid filling wave velocity, e’ = mitral annular early diastolic velocity, CRT = cardiac resincronization therapy, HF = heart failure, LA = left atrium, LBBB = left bundle branch block, LV = left ventricular, MAC = mitral annular calcification, MR = mitral regurgitation, MS = mitral stenosis, MV = mitral valve, RV = right ventricular, TR = tricuspid regurgitation.

Cardiac ultrasound has the unique ability to non-invasively estimate LV filling pressures using a comprehensive, stepwise approach, together with associated structural cardiac abnormalities in patients with unexplained dyspnoea or with clinical manifestations of heart failure, irrespective to the EF (Figure 1).

##### Mitral Inflow Velocities and E/A Ratio

The early rapid filling wave (E) velocity and the late filling wave due to atrial contraction (A) velocity are related to the transmitral pressure gradient. When there is a suspicion of myocardial disease, a mitral flow pattern with E/A ratio >2 due to high E and low A wave peak velocities and short E-wave deceleration time (<160 msec) is a restrictive mitral filling pattern consistent with increased LV filling pressures. If the E/A ratio is ≤0.8 and the E velocity is <50 cm/s, as a consequence of a small transmitral pressure gradient, the filling pressures are considered to be normal (Figure 1). For values in between, three other additional criteria should be applied: E/e’, left atrium (LA) maximum volume index and peak tricuspid regurgitation velocity [20].

##### E/e’ Ratio

E/e’, the ratio between E wave peak velocity and mitral annular early diastolic velocity, e’, is widely used for the estimation of LV filling pressures and it has been shown to correlate with invasive measurements of LV filling pressures [21]. In myocardial diseases, E/e’ increases with rising filling pressures, due to higher mitral E peak velocity, while e’ velocity remains low as a consequence of impaired LV relaxation and minimum elevated LV diastolic pressure. An average E/e’ ≥ 14 is indicative of elevated LV filling pressures [22].

E/e’ has a good correlation with LV filling pressures in patients with HF with preserved EF (HFPEF). However, in patients with HF with reduced EF (HFREF), on resynchronization therapy or with dilated hearts, there is only a moderate correlation between septal E/e’ > 15 and invasively measured LV filling pressures [23,24]. 

##### Peak Tricuspid Regurgitation (TR) Velocity and Systolic Pulmonary Artery Pressure (sPAP) Estimation

The increase in sPAP is another criterion for the estimation LV filling pressures, if other non-cardiac causes of pulmonary hypertension or pulmonary arterial hypertension are excluded. sPAP is calculated as the sum of systolic tricuspid pressure gradient using peak TR velocity and the estimated right atrial pressure, based on the inferior vena cava (IVC) measurement. A peak TR jet velocity >2.8 m/s is strongly indicative of elevated filling pressures. 

However, TR jet cannot be accurately recorded in about half of the patients, mainly with preserved EF [25,26] The short pulmonary artery acceleration time (AccT), less than 100 ms, is an alternative parameter indicative of sPAP elevation. Mean PAP (mPAP) can be estimated using the equation: mPAP = 79 − 0.45 × AccT [27].

Also, in patients with acute pulmonary embolism, right ventricular infarct, or severe right ventricular dysfunction, the markedly increased right atrial pressure will lead to a low peak TR velocity, regardless of left-sided filing pressures.

##### Left Atrial Maximal Volume Index

The LA enlargement is a consequence of the chronic effect of long-term increase in LV filling pressures in patients without atrial fibrillation or flutter, mitral valve disease, bradycardia or high-output states. The maximal LA volume index (LAVI) >34 mL/m^2^ by 2D echocardiography is integrated in the algorithm for the estimation of LV filling pressures and it is associated with higher cardiovascular risk in HFPEF [22,28]. LA enlargement has several limitations: it does not reflect the instantaneous pressure changes, has a low sensitivity for the detection of early elevation of LV filling pressure and LA volume can be increased in highly trained athletes [22,29].

Recently, two large multicenter studies with simultaneous evaluation of LV filling pressures by echocardiography and invasive measurements (LV end-diastolic pressure, pulmonary capillary wedge pressure or LV pre-A pressure) have confirmed the high feasibility and good accuracy of the 2016 recommendations for echocardiographic assessment of LV filling pressure. The algorithm for estimation of LV filling pressure proved to be useful both in patients with unexplained exertional dyspnoea, and in HF patients, regardless of the LV EF [26,30].

##### LA Function

The function of LA as a reservoir, conduit and pump can be assessed by 2D echocardiography using myocardial deformation techniques. LV diastolic dysfunction and increased resistance to filling alter the atrial function before LA dilation occurs. 

In HF, LA peak systolic strain, a marker of atrial reservoir function, has been shown to correlate with the severity of diastolic dysfunction and increased filling pressures. A cut-off of LA peak systolic strain <25% has been proposed for the diagnosis of HFPEF and <18% for the diagnostic of increased filling pressures [31]. LA pump strain, a marker of atrial contraction, is also an useful parameter, a value >14% being indicative of normal filling pressures [20]. Of note, LA peak systolic strain and LA pump strain as standalone parameters, are weakly correlated with LV filling pressures in patients HFPEF and in the general population.

As a consequence, LA reservoir strain was included in the algorithm for LV filling pressure estimation recommended by the ASE/EACVI Guidelines in 2016, if only two of the three additional criteria are available and they are discordant. LA reservoir strain has been proved to have a similar but not superior accuracy to the parameter it substitutes, especially in patients with HFPEF [20].

#### 2.1.2. Right Ventricular Filling Pressure and Systemic Congestion

##### Inferior Vena Cava

IVC diameter (IVCD) with its respiratory changes is a non-invasive parameter widely used for the estimation of right-sided filling pressures and the assessment of changes in intravascular volume [32]. IVC should be measured in the long axis from the subcostal view with the patient either in supine or in semi-recumbent position (when supine position is not tolerated). The IVC size and the collapsibility index (CI) with respiration [CI = IVCD max − IVCD min/IVCD max) × 100] provides information about right atrial pressure, and pulmonary capillary wedge pressure PCWP [33,34]. However, in mechanically ventilated patients, the relationship between right atrial pressure and the IVC size and collapsibility with respiration is weak [35]. 

In normal right atrial pressure, the IVC dimension should be less than 21 mm and the collapsibility index ≥50%. In HF patients with a sustained elevation of right atrial pressure, the IVCD will be larger, and the CI will decrease (<50%). Of note, young persons may have dilated IVC, but the collapsibility index is normal.

As in almost half of HF patients the IVC distension due to increased right atrial pressure was associated with other markers of hemodynamic congestion, despite the absence of symptoms and signs of clinical congestion, the IVC is an important sublinical marker of systemic congestion [36]. 

There are two on-going trials in patients hospitalized for acute decompensated heart failure, in which sequential measurements of IVC are used to monitor and guide decongestion using diuretics in order to reduce re-hospitalization (NCT03140566 and NCT02892227).

##### Doppler Flow in the Hepatic Veins

When right atrial pressure is normal, the Doppler flow pattern in the hepatic veins (HV) has a predominant systolic forward flow. When right atrial pressure becomes elevated, the pressure gradient between HV and right atrium decreases, leading to a HV Doppler flow pattern with a predominant diastolic forward flow. In pulmonary hypertension, due to reduced right ventricular compliance and elevated right ventricular diastolic pressure, the atrial flow reversal is markedly increased and has little respiratory variation. In severe tricuspid regurgitation there are systolic flow reversals in the hepatic veins. The assessment of respiratory changes of Doppler flow patterns in the HV is useful to differentiate restrictive cardiomyopathy from constrictive pericarditis.

##### Ultrasonographic Evaluation of Intrarenal Venous Flow

The assessment of hemodynamic changes due to systemic congestion in HF on the intrarenal venous flow (IRVF) patterns may provide important information about the renal congestion severity, the consequences on renal function and the reversibility of abnormal IRVF patterns after de-congestion. The IRVF can be assessed by pulsed wave Doppler flow of the interlobar arteries and veins, with concomitant recording of the arterial (above the baseline) and the venous (below the baseline) Doppler signals. 

The normal IRVF is continuous during the cardiac cycle. As the right atrial pressure increases, there are progressive changes in the venous flow, from discontinuous, “pulsatile” flow to biphasic and even monophasic flow (the velocity progressively diminishes during systole, and the flow occurrs only during diastole in patients with very high right atrial pressure) [37].

The qualitative analysis of IVRF patterns can be completed by quantitative analysis, using the venous impedance index (VII) and renal venous stasis index (RVSI). VII is calculated using the formula [(maximum velocity-minimum velocity)/maximum velocity], and RVSI is defined as the ratio between duration of absent venous flow divided by the duration of the cardiac cycle.

The changes in IRVF patterns reflect the elevated right-side pressures transmitted backward, leading to increased interstitial and tubular hydrostatic pressure within the encapsulated kidney and lowering the glomerular filtration rate. The parenchymal vessels are compressed, and the venous flow becomes pulsatile, being dependent on the cardiac cycle, due to increased compliance of the congestive renal parenchyma. There are similar changes in hepatic venous flow [38,39]. 

In a recent study, in several patients with stable chronic HF the IVRF pattern became discontinuous after volume loading, even without change in IVC size, suggesting that changes in IVRF pattern could be an earlier index of systemic congestion [40]. 

More recent data bring important evidence supporting the use of IRVF pattern as a congestion marker. Under decongestive therapy the normalization of the discontinuous IRVF patterns occurs in approximately one-half the patients hospitalized with decompensated HF, most of them (87%) showing impaired IRVF patterns at admission. No changes in the IRVF patterns were noted in patients with renal dysfunction at baseline [41]. 

Serial evaluation of IRVF patterns in stable ambulatory HF patients demonstrated that discontinuous patterns during the follow-up period, rather than those at baseline, were associated with poor outcomes (cardiovascular death and HF hospitalization). Moreover, persistent discontinuous pattern was associated with renal impairment progression [42]. 

The abnormal IVRF patterns are non-specific markers of systemic congestion, as it may occur in other conditions associated with increased pressure in the renal parenchyma, such as obstructive uropathy, or increased intra-abdominal pressure [43].

##### Ultrasonographic Evaluation of the Internal Jugular Vein

The clinical examination of neck veins for the assessment of central venous pressure in right ventricular failure if often limited in obese patients and has an intra- and intraobserver variation. The evaluation of the internal jugular vein (IJV) compliance using ultrasound could be a useful parameter for the assessment of systemic congestion. Due to the curvilinear shape of the relationship between intravenous pressure and volume, the venous compliance is high at low venous pressures and decreases when the venous pressures become elevated. 

The dimension of IJV should be measured at rest (at end-expiration) and during Valsalva manoeuvre, which acutely increases the right atrial pressure. When right atrial pressure is normal, there is a substantial increase in IJV dimension during a Valsalva manoeuvre. 

For the measurement of the dynamic changes of IJV two methods can be used: the diameter and cross-sectional area of the vein. The JV distention (JVD) ratio is calculated as the ratio between the maximal diameter during Valsalva manoeuvre to the diameter at rest [44]. Since the maximal diameter of IJV during Valsalva manoeuvre is the same regardless the presence of HF, while the IJV diameter at rest is increased in patients with systemic congestion, the JVD ratio is reduced. A JVD ratio <4 is considered abnormal and the ratio may decrease <2 in severe congestion [45].

The cross-sectional area of IJV will increase by 20–30% during Valsalva manoeuvre in patients with normal right atrial pressure. An increase in IJV cross sectional area >17% during Valsalva manoeuvre effectively ruled out patients with elevated right atrial pressure invasively measured by right heart catheterization [46,47]. 

The evaluation of congestion using the IJV compliance by ultrasound is limited in patients unable to perform a Valsalva manoeuvre.

## 3. Imaging of Pulmonary Congestion

The overt complete clinical picture of pulmonary congestion is a late manifestation of interstitial and alveolar oedema. However, in most instances, in patients with imminent acute HF, there is a transition subclinical period in which a gradual accumulation of extravascular water occurs [48]. Identifying subclinical pulmonary congestion offers a diagnostic advantage as well as a therapeutic window of opportunity, in which timely decongestive therapy can reduce the progression to overt symptomatic HF [49]. Moreover, complete pulmonary decongestion at hospital discharge is associated with an improved prognosis [50]. Therefore, the past years have seen a continuous search for a biomarker that can reliably identify and quantify subclinical pulmonary congestion. 

Several clinical, imaging (radiological, ultrasound) and non-imaging methods have been proposed in order to depict and quantify increased lung water.

### 3.1. Chest Radiography and Computer Tomography 

Chest radiography (CxR) is a widely available and inexpensive method for assessing causes of dyspnoea especially in the emergency room [51]. A sequence of radiological signs can be described as the severity of pulmonary congestion increases-starting with vascular “redistribution” towards the upper lobes followed by enlargement of the vascular pedicle, Kerley A and B lines, thickening of interlobar fissures and pleural effusion. The most extreme form of pulmonary congestion, acute pulmonary edema, will manifest as bilateral and usually symmetric alveolar opacities, with a central distribution [52]. However, CxR has a sensitivity of only 57% (95%, confidence interval 55–59%), and a specificity of 89% for a diagnosis of pulmonary oedema [53]. Moreover, CxR is associated with radiation exposure, high inter-observer variability and depicts only the more advanced forms of pulmonary congestion [54]. 

Thoracic computer tomography can identify pulmonary fluid accumulation however, its use as a marker of pulmonary congestion in the very prevalent HF population is refrained due to cost, availability, and radiation exposure [51]. 

Intrathoracic impedance falls as the amount of fluid in the lungs increases therefore, implantable or non-invasive lung impedance monitors have been proposed for assessing pulmonary congestion and guiding HF therapy [54,55]. However, conflicting results have been published and currently these devices are inadequately validated [55].

### 3.2. Lung Ultrasound

In the past 10 years, lung ultrasound (LUS) has emerged as a non-invasive imaging biomarker of pulmonary congestion with diagnostic, monitoring, therapeutic and prognostic value in the management of HF patients [49,51,56,57,58,59]. 

LUS behaves as a densitometer accurately evaluating the progressive decrease in lung aeration [49,58]. In a normally aerated lung a normal lung pattern can be described as the presence of lung sliding and A lines [57,60]. However, as the air content of the lung decreases due to increased interstitial or alveolar accumulation of transudate or exudate, vertical artifacts called B lines will be visible [61]. The total loss of parenchymal aeration will create a consolidation pattern resembling that of a solid organ such as the liver [62]. Pleural effusion is also readily seen on LUS as an anechoic structure between the pleura and the lung parenchyma [61].

#### 3.2.1. The Role in HF Diagnosis

LUS is a quantitative, simple and rapid method of assessing lung congestion. The main diagnostic findings suggestive of interstitial syndrome (caused by increased extravascular transudate or exudate) are the B lines [57,60]. These are vertical hyperechoic laser-like artefacts that arise from the pleural line and extend to the bottom of the screen without fading, moving in sync with lung sliding, and erasing A lines [50].

Evaluation of the presence and number of B lines on LUS as a marker of interstitial syndrome has shown good correlation with clinical, radiological, invasive, and post-mortem markers of lung congestion and has shown prognostic and therapeutic value [48]. 

**Figure 2:**
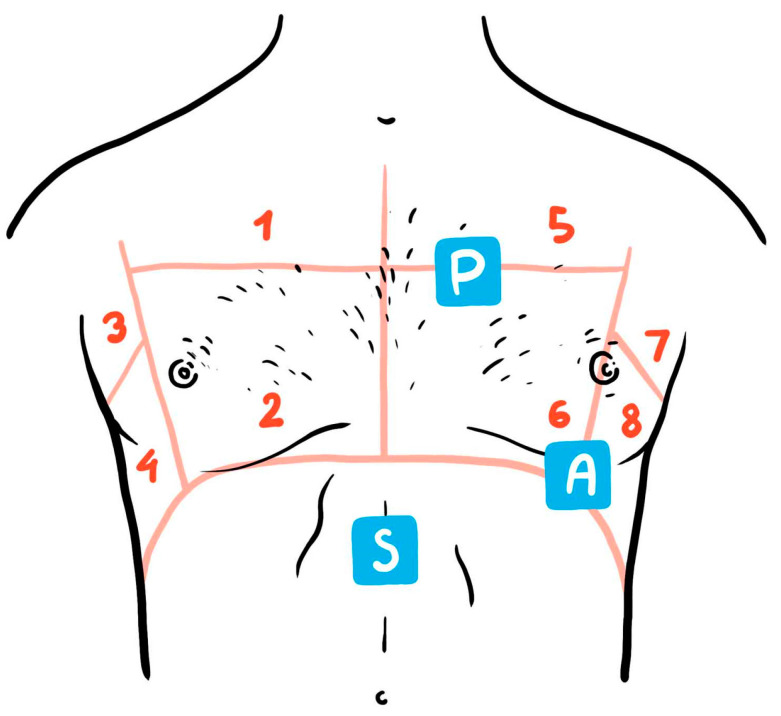
Lung UltraSound (LUS) applying the eight chest zone method and the Point-of-care Cardiac UltraSound examination (FoCUS) with the main examination windows (P–parasternal long and short axis views; A-apical four-chamber view; S-subcostal views of inferior vena cava and cardiac four-chamber) [53].

B lines are very useful in establishing the cardiac cause of acute dyspnoea in the emergency room [51,58]. Data from a recent randomized trial showed that a strategy combining LUS with clinical assessment has a higher diagnostic accuracy and reduces diagnostic errors when compared with the use of CxR and N-terminal proBNP (NT-proBNP) in conjunction with clinical evaluation, in establishing the correct diagnosis of acute HF. Moreover, the LUS strategy significantly reduces the time necessary to formulate the correct diagnosis [63]. LUS was shown to be more sensitive than CxR in detecting pulmonary edema in acute HF patients [64]. The presence of B lines can differentiate acute HF from non-cardiac causes of dyspnoea with a sensitivity of 94% and a specificity of 92% as reported in a large meta-analysis [65]. Accordingly, LUS has developed as a useful adjunctive point-of-care tool in emergency for the differential diagnosis of acute dyspnoea and hemodynamic instability along with FoCUS [58] (Figure 2). In the emergency setting, the absence of a “wet lung” pattern–multiple, diffuse, bilateral B lines-on LUS excludes cardiogenic acute pulmonary edema with a very high negative predictive value [59].

#### 3.2.2. The Role in Monitoring Congestion

The B lines assessed with LUS are dynamic and their number and location can change rapidly after decongestive therapy making them an attractive biomarker that could help in the management of pulmonary congestion [66]. Acute changes in B lines were demonstrated in patients with HF after diuretic therapy, in patients treated for hypertensive emergencies as well as in patients undergoing hemodialysis. Moreover, a rapid increase in B lines was demonstrated with exercise in both HFPEF and HFREF patients during stress echocardiography [64,65]. The number of stress induced B lines as a marker of pulmonary congestion correlated well with brain-type natriuretic peptide (BNP) value, echographic parameters of systolic and diastolic function and their presence were associated with a greater functional impairment [67,68,69]. 

Therefore, B lines assessment is a promising marker of pulmonary congestion and decongestion. In a single center small trial, LUS helped in tailoring diuretic therapy of patients with acute HF and was able to accelerate the discharge time [70]. Three recent randomized trials assessed the usefulness of LUS guided decongestive therapy in HF in acute and chronic settings [71,72,73]. The BLUSHED-AHF (B-lines Lung Ultrasound–Guided ED Management of Acute Heart Failure Pilot Trial) was a multicenter, single-blind, emergency department-based, pilot trial which randomized 130 patients to receive a 6-h LUS-guided treatment strategy versus structured usual care. In this trial, LUS guided strategy conferred no benefit compared with standard care in reducing the number of B-lines at 6 h or in improving prognosis. However it did help in reducing congestion faster during the first 48 h [72]. The LUS-HF trial was a single centre, single-blind, clinical trial that randomized 123 recently discharged HF patients to LUS-guided follow-up or standard follow up. Diuretic therapy was changed according to the number of B lines on LUS. LUS-guided strategy significantly improved the combined endpoint of urgent visit, hospitalization for worsening HF and death during a 6-month follow-up in patients after HF admission. Moreover, a reduced number of clinical decompensations and an improved walking capacity ware observed [73]. CLUSTER-HF, which followed a similar trial protocol to LUS-HF, showed also that a LUS-guided follow-up of HF patients results in a reduction of urgent visits for worsening HF [71]. 

Other biomarker-guided strategies have been evaluated in the follow-up and management of HF patients. However, the main advantage of the LUS strategy is its non-invasiveness and the negligible associated cost [74,75,76]. 

#### 3.2.3. The Prognostic Role

Pulmonary congestion as reflected by B lines assessed with LUS has an important prognostic role in ambulatory or admitted HF patients [57,77]. The EVEREST trial showed that clinical congestion in HF patients during hospitalization and at discharge is associated with worse prognosis. However, even patients judged to be congestion free at discharge have a comparative poor prognosis [78]. Therefore, subclinical congestion conveys a poor outcome and needs to be identified. LUS has emerged as a robust imaging marker for lung congestion and can identify patients in the pre-clinical stages of pulmonary congestion [48]. 

In hospitalized HF patients, LUS-detected pulmonary congestion was found to be an independent predictor for short-term mortality and HF hospitalization [79]. The cut-off B lines number to identify patients with worse outcome is still to be established. In one study, the most appropriate B line cut-off varied according to the time of assessment with B-lines ≥45 for the early phase of HF hospitalization and B-lines ≥30 at discharge [79,80]. Another study from the same group was able to confirm the prognostic value of residual congestion at discharge evaluated by LUS in HF patients. A residual B line count ≥30 was a strong predictor of outcome [80]. Moreover, the presence of residual ultrasonographic lung congestion at discharge is strongly associated with HF hospitalizations at 6 months and a B lines count ≤15 identifies a subgroup of patients at extremely low risk to be readmitted for HF decompensation [50]. Furthermore, an increase of 1 point in the B lines sonographic lung score is associated with a 24% increase in the risk of HF hospitalization and death at 100 days [81]. 

The prognostic value of LUS is preserved in ambulatory, chronic, HF patients. It was shown that a higher number of B-lines on LUS identified patients at risk for urgent HF visits, HF hospitalizations, or death from any cause over 6 months, independent of age, sex, NYHA class, and clinical congestion score [82]. 

LUS is an important prognostic marker both at rest as well as when assessed during stress. B lines development or worsening during exercise is associated with higher levels of natriuretic peptides at baseline, higher pulmonary artery pressures, worse functional impairment and poor outcome [67,68,69]. A number of ≥10 B lines at peak stress were identified as an independent predictor of death and myocardial infarction and an increasing number of B lines at peak stress were associated with progressively worse event-free survival [68]. 

In end stage chronic kidney disease patients undergoing dialysis, subclinical pulmonary congestion is frequent. LUS is able to detect increased lung water content which was shown to predict poor outcome [83]. Patients with very severe congestion (>60 B lines) have an adjusted 4.2-fold risk of death and a 3.2-fold risk of cardiac events [84]. 

#### 3.2.4. Limitations

LUS is a very sensitive technique for assessing lung congestions however B lines are not specific for cardiogenic pulmonary edema. B lines appear in any pulmonary interstitial syndrome such as interstitial pneumonia, acute respiratory distress syndrome, COVID-19 lung disease interstitial fibrosis [57,85]. A separation between these clinical syndromes requires the integration of multiple clinical, laboratory and imaging parameters [58,59,86]. 

LUS is a very feasible echographic technique even though it can be challenging to obtain acceptable image quality in patients with morbid obesity, subcutaneous emphysema, soft tissue edema or large wound dressings [56]. 

LUS is a near-ideal imaging marker of lung congestion and its value it’s complemented by additional clinical, laboratory and imaging markers. Its inclusion in a multiparametric congestion score will likely improve the diagnostic and prognostic value. As such, currently, the Heart Failure Association of the European Society of Cardiology recommends an integrative evaluation of congestion in HF patients including LUS [10].

The most commonly used clinical scores and the cardiac, vascular and pulmonary ultrasound parameters of congestion in HF are depicted in Table 1.

## 4. Biomarkers

Biomarkers can be measured objectively as indicators of physiological or pathogenic processes, as well as responses to therapeutic interventions, with no inter-observer variability [92]. Several biomarkers have been studied as early diagnostic tools, prognostic indicators, or therapeutic response estimates in the setting of heart failure.

### 4.1. Natriuretic Peptides

NPs have been discovered few decades ago and extensively studied thereafter, proving to be the gold standard biomarkers for the diagnosis, prognosis, monitoring and treatment in patients with HF. NPs have also added prognostic value for HF risk screening in other cardiovascular diseases.

BNP and atrial-type natriuretic peptide (ANP) are neurohormones synthesized and released into the bloodstream exclusively by ventricular and atrial cardiomyocytes in response to increased end-diastolic wall-stress due to volume or pressure overload. NPs are synthesized as pre-prohormones, then cleaved as prohormones and subsequently cleaved into active hormones, BNP and ANP, and the inactive NT-proBNP and mid-regional proANP (MR-proANP). BNP and ANP play adaptive counter-regulatory roles, by binding to NP-receptors at several levels and causing vasodilation, natriuresis and diuresis. BNP and ANP have short circulating half- lives (20 min, respectively 2–4 min), being actively cleared from the circulation due to NP receptors and plasma endopeptidases. NT- proBNP si MR-proANP have longer circulating half-lives (around 90 min) and their clearance is primarily renal. They are useful molecular targets for biochemical measurement, being used as surrogates for plasma levels of BNP and ANP [93,94].

Although NP are continuous variables, the heart failure guidelines currently recommend cut-off values of NP plasma concentration to “rule-out” or “rule-in” the diagnosis of HF in patients with dyspnoea and clinical suspicion of HF [95]. BNP and NT-proBNP have high sensitivity for excluding HF. The higher the NP level, the probability of HF increases, due to a proportional NP release by the failing heart as a response to the increased wall stress. Therefore, circulating NP levels mirror the severity of the hemodynamic abnormality, meaning wall stress, the product of intracardiac volumes and filling pressures and thus the severity of the congestion [96]. However, the use the NP cut-off values is more challenging to “rule-in” HF, due to the complex influence of many factors, such as age, renal function, obesity, atrial fibrillation, acute vs chronic HF, HFREF vs HFPEF and the non-linear relationship between NP levels and disease severity [97] (Table 2). Higher levels of NP have been observed in women compared to men in healthy population. However, these gender-related differences are lessened in patients with HF, and current guidelines do not recommend the use of different cut-offs for men and women [98].

The value of BNP and NT-proBNP as biomarkers have been more extensively investigated than ANP and MR-proANP. However, MR-proANP proved to be a more sensitive and specific and also a prognostic biomarker of HFPEF than NT-proBNP, as high plasma levels of MR-proANP were associated with increased 10-year all-cause mortality in patients with HF [99,100,101]. 

NPs might be able to detect very early stages of congestion in HF and may estimate risk of readmission [102]. The decrease of NP levels before discharge in patients with congestion reflect haemodynamic improvement, with some evidence of a better survival in patients with a marked NP decrease [103,104]. However, more data are needed regarding NP level at hospital discharge that could be associated with euvolemia, with improvement of prognostic and with lower risk of rehospitalisation. Conversely, an increase in BNP plasma levels even without clinical signs of congestion was associated with an increased rate of rehospitalisation for HF [105]. 

In HF patients, the de-congestive treatment with diuretics and the long-term treatment with beta-blockers, renin-angiotensin-aldosterone system inhibitors and cardiac resynchronization therapy, due to their beneficial effects on cardiac remodelling and reducing ventricular wall stress, would lead to a reduction in NP concentrations. Of note, the plasma levels of NP would transitorily increase during early treatment with non-vasodilator beta-blockers. The treatment with sacubitril-valsartan, due to the neprilysin inhibition, also tends to increase the BNP levels in HF patients, especially in those with higher baseline values [10].

In summary, NP values are very useful tools for assessing the congestion in HF patients, but always in relation with clinical data. Their inherent limitations stimulated further research aimed to validate other biological and imaging markers of congestion. 

**Table 2:** Natriuretic peptides cut-off values in different heart failure setting.

HF Clinical Setting	NT-ProBNP (pg/mL)		BNP (pg/mL)		MR-ProANP (pg/mL)	Comments
	Rule-In	Rule-Out	Rule-In	Rule-Out	Rule-Out	
Suspected acute HF (Patients with acute dyspnoea) *	Age–related <50 y **>450** 51–75 y **>900** >75 y **>1800**	**<300**	**>400**	**<100**	**<120**	Higher NP levels in HFREF vs HFPEF [13] Less data for MR-proANP
Suspected acute HF and eGFR < 60 mL/min	Same as in suspected Acute HF			<200	-	No supplementary correction recommended for NT-proBNP age-adjusted cut-offs due to correspondence between renal function decline and increasing age [13]
Suspected acute HF and AF	>600 (SOCRATES trial [106]) >900 (PARAGON trial [107])	<400	>240	<150	-	Higher NP levels occasionally observed in patients with AF but no clinical data to sustain HF diagnosis [108] In HFPEF trials, the NP cut-off values as inclusion criteria were higher in patients with AF vs sinus rhythm [106,107]
Suspected acute HF and obesity > 30 kg/m^2^	Lowering the cut-off levels by up to 50%			<50	-	Presumed overlap between NP levels in HFPEF and obesity [109]
HF in the community (Non-acute setting)	>600	<125	>150	<35	<40	NP serial dosing during follow-up in conjunction with symptoms and weight gain are recommended in order to recognize early decompensation [13].

AF = atrial fibrillation; BNP = B-type natriuretic peptide; Egfr = estimated glomerular filtration rate; HF = heart failure; HFPEF = heart failure with preserved ejection fraction; HFREF = heart failure with reduced ejection fraction; MR-proANP = Mid-regional pro-atrial natriuretic peptide; NP = natriuretic peptide; NT-proBNP = N-terminal pro-B-type natriuretic peptide; * During HF hospitalization the lack of decrease/ any increase and, during the follow-up visits an increase more than 50% of NP value is likely to be of clinical relevance of increased filling pressures [13]. NT-proBNP <1500 pg/mL or ≥30% decrease/BNP <250 pg/mL under treatment at discharge is considered a favorable NPs change in HFREF patients [7], although other data sustain a greater benefit when lower target NP concentration is attempted (BNP *<* 100 pg/mL, NT-proBNP *<* 1000 pg/mL) [110].

### 4.2. Cardiac Troponin: Conventional and High-Sensitivity Assays

Cardiac troponins (cTn) are routinely measured in patients with acute HF in order to rule out an acute coronary syndrome. Actually, cTn are often elevated in patients with acute HF due to acute or chronic cardiomyocyte death or injury in the absence of ischemia, possibly caused among other mechanisms by elevated intracardiac filling pressures, increased wall stress, subendocardial ischemia [111,112]. Congestion on admission was significantly associated with cTn levels at discharge, implying that the increased wall stress accompanying HF decompensation may induce subclinical myocardial injury [113]. 

When measured with a conventional assay, elevated cTn in the absence of a type 1 myocardial infarction on admission for acute HF associates an increased risk of death during hospitalization or post discharge and also for HF readmission [114]. Moreover, serial assessment of cTn during hospitalization for acute HF provided prognostic value. Patients with a positive cTn level on admission or during hospitalization had higher mortality and readmissions than patients with negative cTn throughout hospitalization [115]. 

High sensitivity cardiac troponin (hs-cTn) assays have also been proven useful for prognosis in patients with acute HF. Very low levels of hs-cTn predict a good prognosis whereas any elevation is associated with an increased risk of death or HF readmission [116]. Serial monitoring of hs-cTn showed that both higher baseline and peak hs-cTn values were associated with higher risk of cardiovascular mortality and HF hospitalization [117]. 

Besides their utility in prognostic evaluation, cTns may be as well useful in guiding therapy in acute HF. Several studies demonstrated that cTn levels change with adequately applied medical therapy. The use of angiotensin converting enzyme-inhibitors (ACEIs) and angiotensin receptor blockers was associated with undetectable levels of cTn, whilst the absence of beta-blocker use was associated with higher levels of cTn [118]. Treatment with an angiotensin receptor-neprilysin inhibitor determined a significant and sustained reduction in hs-cTn compared to treatment with ACEI alone [119]. 

### 4.3. Emerging Congestion Biomarkers of Cardiac Origin

#### 4.3.1. Soluble Suppression of Tumorigenesis-2

Although initially classified as a marker of myocyte stress, the expression of soluble suppression of tumorigenesis-2 (sST2), the circulating form of the interleukin-33 membrane receptor, is largely a response to vascular congestion, mechanical stretch and to inflammatory and pro-fibrotic stimuli in extracardiac tissues [120,121,122]. The lungs have been documented as a relevant source of sST2 in HF, but the sites of sST2 proteolysis and the specific proteases have not been clarified [123]. 

Cardinal requirements for clinically useful biomarkers are met by sST2, providing information additive to a thorough clinical examination, complementing the prognostic value of natriuretic peptides and, in chronic HF, to hs-cTn T [122]. The commonly used cut-off of 35 ng/mL makes sST2 a powerful predictor of mortality and hospitalization in acute or chronic HF independently of NT-proBNP, hs-troponin T, and LVEF, almost unaffected by age, sex, body mass index, renal function or ischemic aetiology [120,124]. Moreover, NPs, hs-cTn and sST2 mirror distinct HF pathways, enhancing risk prediction beyond echocardiographic LVEF, and all these predictors have been included in the Barcelona Bio-HF Calculator Version 2.0, along with clinical variables and HF treatment, predicting all-cause mortality, HF-related hospitalization and the composite of these endpoints for five years [118]. sST2 is also a parameter in the ST2-R2 score for the likelihood of reverse remodelling in HFREF, alongside other markers [120]. 

High levels of sST2, as a biomarker of fibrosis, have been linked to a higher risk of sudden cardiac death, therefore the decision for an ICD could be based on them [123]. In right ventricular dysfunction and pulmonary hypertension, the roles of sST2, troponin, galectin-3 and growth differentiation factor-15, as well as advanced imaging require further clarification [120].

The limitations of using sST2 as a cardiovascular diagnostic marker include its vulnerability to concomitant inflammation, such as autoimmune diseases, liver failure, asthma, sepsis [125,126]. The method to measure sST2 has been another open question, but a recently validated automated turbidimetric immunoassay for ST2 may boost sST2 use in everyday practice [127]. The best moment to measure sST2 levels in HF is still a matter of debate, but in an acute setting it should be assessed at least on admission and discharge. In chronic HF it could be a tool for risk stratification, perhaps even for guiding therapy, alone or in combination with NP and troponins, but this merits further investigation [124].

Overall, while sST2 is a powerful outcome predictor, the question remains whether a sST2-guided management of acute or chronic HF could optimize disease progression and symptoms.

#### 4.3.2. Carbohydrate Antigen 125 (CA125)

Carbohydrate antigen 125 glycoprotein has been used for a long time as biomarker for ovarian cancer surveillance. Recent evidence showed CA125 as a possible marker of congestion of cardiac origin, as it was positively correlated with fluid overload, increased atrial and pulmonary pressure, right-ventricular dysfunction, and also with the risk of adverse events [128]. 

#### 4.3.3. Soluble Melanoma Cell Adhesion Molecule (CD146)

Soluble CD146, known as a marker of endothelial function, was linearly correlated to the severity of pulmonary congestion evaluated by chest radiography, and also to LV function and congestion in acute HF [129,130].

#### 4.3.4. Mid-Regional Pro-Adrenomedullin (MR-ProADM)

Adrenomedullin acts as a circulating hormone but has also local autocrine and paracrine actions with vasodilatory and natriuretic properties within cardiovascular, renal, pulmonary, cerebrovascular, gastrointestinal, and endocrine organs [131]. 

It has proved prognostic value, superior to both BNP and NT-proBNP in predicting 14-day mortality in acute setting and additive to BNP and NT-pro BNP in predicting 90-day mortality [100,132]. Experimentally data showed significantly higher ADM plasma concentration in pulmonary congestion than in compensated HF [132]. 

### 4.4. Other Biomarkers of Systemic Congestion

Elevated central venous pressure in HF may contribute to worsening kidney and liver function. Abnormalities in liver and kidney function tests are common findings in patients with HF, with an incidence varying between 25% and 75% [133].

#### 4.4.1. Renal Function Biomarkers

Venous congestion has been shown to be the most important hemodynamic contributor to worsening renal function in patients with decompensated HF patients. Therefore, during hospitalization the blood urea nitrogen to creatinine ratio can be useful as a marker of congestion or it may suggest decongestion [24,134]. Although there are several criteria, an increase in serum creatinine ≥0.3 mg/dL during hospitalization could be a useful marker of either worsening of renal function (WRF) without kidney injury, or acute kidney injury (AKI). AKI occurs particularly in patients with baseline chronic kidney disease having a decline in urinary output associated with biochemical markers of intrinsic renal injury on urine microscopy [135]. WRF during HF hospitalization could reflect an intensive de-congestion with dehydration, renal hypoperfusion due to low cardiac output or intensive treatment with diuretics and Renin-Angiotensin-Aldosterone-System (RAAS) inhibitors [136]. The prognosis of WRF in HF patients should be considered in relation to its severity, duration and, in particular, to the congestion status. A transient increase in serum creatinine associated with increased diuresis, initiation of RAAS inhibitors and, notably, effective de-congestion did not predict a worse prognosis. However, the persistence of both congestion and WRF is associated with worsened prognosis [137]. 

#### 4.4.2. Liver Function Biomarkers

Increased central venous pressure with subsequent high right atrial pressure transmitted to the hepatic veins, may alter hepatocyte function, resulting in cholestatic liver injury [138]. Moreover, decreased cardiac output with subsequent low liver perfusion may induce acute hepatocellular necrosis [139]. Therefore, bilirubin, alkaline phosphatase and gamma-glutamyl transpeptidase have been suggested as possible biomarkers for congestion [7]. Elevated levels of aspartate aminotransferase, alanine aminotransferase, and bilirubin are found in patients with low cardiac output [140]. Moreover, elevated alkaline phosphatase and transaminases correlate with increased mortality risk [141]. 

#### 4.4.3. Haemoconcentration

Plasma volume may be indirectly estimated by several formulas using haemoglobin and/or haematocrit level, which seem useful for monitoring congestion and decongestion both in acute in chronic setting. Plasma volume variation can be evaluated using Strauss formula: % change in plasma volume = 100 × haemoglobin before/haemoglobin after × (1 − haematocrit before)/(1 − haematocrit after) − 100. Instantaneous plasma volume estimation can be calculated using Duarte’s formula: 100 × (1 − hematocrit)/hemoglobin [142]. 

Several other biological parameters that are routinely determined in patients with HF, such as serum protein, albumin, hemoglobin, and hematocrit have been associated with prognosis, being proposed as alternate markers for monitoring congestion. However, their utility as de-congestion biomarkers is limited by the fact that small changes may be caused by other HF-associated conditions and also they do not reflect the absolute change in plasma volume [143].

### 4.5. Point of Care Testing

The value of biomarkers in HF management is strengthened by their availability in a short time, which became possible through the Point-of-Care Testing (POCT) devices development. POCT can be performed next to or very close to the patient’s bed, both sample collection and analysis, testing results being available within minutes (about 20 min). Besides this remarkable time advantage as sample transport and processing are not necessary, compared to conventional testing, requiring centralized laboratory settings and trained personnel, POCT can be very easily performed by any user. Moreover, POCT devices do not necessitate sophisticated equipment, the technique being simplified due to advancements in the area of microfluidics and nanotechnology [144]. Hence another advantage of POCT devices, portability, providing the possibility to perform testing on an outpatient basis, which make them excellent screening as well as serial testing tools [145]. 

Identification of biomarkers in cardiovascular disease through POCT has been available for many years, the first implemented technique delivering qualitative results. Although POCT for cardiac troponin measurement is available for twenty years, the performance of most commercial devices is far beyond the high sensitivity assays performed within the central laboratories [146]. Currently, troponin assays are performed using POCT in patients presenting with chest pain for the early rule-out of myocardial infarction. The use of POCT hs-cTn to stratify the risk in patients with HF appears as a promising perspective, yet randomized clinical trial data is lacking [147].

On the contrary, POCT devices can determine NP levels just as accurate as central laboratory platforms [148]. Most POCT devices perform biomarker analysis using blood samples, but recently the possibility to detect salivary biomarkers became an emerging area of research. Salivary diagnostic appears promising in monitoring patients with HF, as collection process is non-invasive and several useful biomarkers can be detected, among which BNP, NT-proBNP, cTnI [149]. 

Although POCT systems offer several advantages to healthcare, there are limitations to their widespread use in clinical practice: the discrepancies between POCT and central laboratory results, as well as between different POCT models, lack of appropriate training for test performing and device maintenance, economic and local infrastructure issues that delay the implementation of these devices [150,151].

Thereby, there are limited data from studies supporting the role of point-of-care NP serial testing in ambulatory care as a tool for monitoring patients with HF and there are no established guidelines recommendations in this regard. The POC-HF (Point-of-Care in heart failure) is a pilot study conceived to demonstrate the usefulness of serial NT-proBNP measurements using POCT in patients hospitalized for acute decompensation of HF [152,153]. 

However, larger prospective trials should confirm the possible positive results of this study. Presumably, POCT sustained by clinical examination and ultrasound imaging might improve the management of patients with HF in ambulatory setting.

## 5. Conclusions

Congestion, the most common cause of HF decompensation, is responsible for recurrent hospitalizations, accelerating disease progression and worsening the prognosis. Therefore, the evaluation of the congestion severity, monitoring its course during treatment, and early detection of subclinical congestion after discharge are important targets of the management of patients with heart failure. In addition to conventional imaging tools, among which echocardiography plays a central role, more and more studies sustain the advantages of lung ultrasound in assessing pulmonary congestion and de-congestion. Complementary to natriuretic peptides, the gold standard biomarkers for the diagnosis, prognosis, and treatment monitoring in patients with heart failure, a large amount of evidence supports the value of several other biochemical markers for the diagnosis of congestion 

In summary, the evaluation of congestion in HF implies a comprehensive, multiparametric approach using imaging and circulating biomarkers in different algorithms adapted to the clinical setting of the patient with heart failure.

## Figures and Tables

**Table 1:** Clinical and ultrasound parameters of congestion in heart failure.

	Clinical Scores/US Parameter	Interpretation	Comments
Clinical examination (Symptoms and signs)	EVEREST * Composite Congestion Score [78] (CCS)	Discharge from HF hospitalization: target CCS ≤ 2	CCS ≥ 3: 10% increase in all-cause death at 6 months
	Lucas Score ^#^ [87]	Severity of congestion at 4–6 weeks after discharge from HF hospitalization	The 2 years mortality: score 0: 13% score 1–2: 33% score 3–5: 59%
Ultrasound			
Lung US	Presence of B-lines (on 8 chest zones)	≥3 B-lines in ≥2 zones/hemithorax identifies acute HF patients (high sensitivity and specificity) [63] Target at discharge <30 B lines [80]	≤15 B lines at discharge: low risk of rehospitalization for HF [50]
Cardiovascular US			
Cardiac	Left atrium Other morphological data	LAVI > 34 mL/m^2^	Reflects long term increase in filling pressures [14,15] In AF or significant MV disease the LA is dilated despite normal LV diastolic function [16]
	Doppler measurements (Algorithm based on the level of EF)	Restrictive mitral filling pattern with the E-wave deceleration time <140 ms	Consistent with increased LV filling pressures in patients with reduced EF [88]
		E/e’ > 15 (and septal e’ < 7 cm/s)	Moderate correlation with invasively measured LV filling pressures in patients with dilated ventricles and reduced EF [24]
		TR jet peak velocity > 2.8 m/s with sPAP > 35 mm Hg	Strongly indicative of elevated filling pressures in the absence of pulmonary disease [22]
Vascular	Inferior vena cava dimension and collapsibility	Max Diam >2.1 cm with Collapsibility index <50% = sustained elevation of RAP	Target at discharge: Max Diam <2.1 cm with Collapsibility index <50% [89]
	Doppler hepatic venous flow pattern	S < D and increased atrial flow reversal = increased RAP [90]	Reversal with effective decongestive therapy [91]
	Doppler intrarenal venous flow pattern	Discontinuous (pulsatile, biphasic or monophasic) = high RAP [39]	Early index of systemic congestion [42]
	Ratio of internal JVDiam during Valsalva manoeuvre	JVDiam ratio <4 in congestion [44]	JVDiam ratio <2 in severe congestion [44]

* EVEREST Composite Congestion Score [78] (CCS) is calculated by summing the individual scores on a standardized 4-points, ranging from 0 to 3 grading scale of severity for dyspnoea, orthopnoea, fatigue, rales, pedal oedema and jugular venous distension. ^#^ Lucas Score [87] is calculated by summing the individual scores on a standardized 2-points ranging from 0 to 1 grading scale of severity for orthopnoea, jugular venous distension, oedema and other clinical parameters: Interpretation: 0 points = no congestion; 1–2 points = mild congestion; 3–5 points = major congestion. AF = atrial fibrillation, CCS = Composite Congestion Score, D = diastolic, Diam = diameter, E = Early rapid filling wave on pulsed wave transmitral Doppler ultrasound, e’ = mitral annular velocities during early diastole, EF = ejection fraction, HF = heart failure, JVD = jugular venous distension, JVDiam = jugular vein diameter, LA = left atrium, LAVI = left atrium volume index, LV= left ventricular, MV = mitral valve, RAP = right atrial pressure, S = systolic, Spap = systolic pulmonary artery pressure, TR = tricuspid regurgitation, US = ultrasound.
